# Évaluation de la prise en charge de l'hypertension artérielle après un accident vasculaire cérébral dans un service de neurologie en Côte-d'Ivoire

**DOI:** 10.48327/mtsi.v4i1.2024.366

**Published:** 2024-01-16

**Authors:** Any GNAZÉGBO, Hiénéya Armel KARIDIOULA, Assata SYLLA, Kotchi Élysée BONY, Yannick Thibaut KOFFI, Aïcha TOURÉ, Bah Abdoul Kader KONÉ, Ange-Éric KOUAMÉASSOUAN

**Affiliations:** Service de neurologie, Centre hospitalier universitaire de Bouaké, Côte d'Ivoire

**Keywords:** AVC, Accident vasculaire cérébral, HTA, Hypertension artérielle, Côte d'Ivoire, Afrique subsaharienne, Stroke, Blood hypertension, Côte d'Ivoire, Sub-Saharan Africa

## Abstract

**Introduction:**

Le contrôle de la pression artérielle est important dans la prévention secondaire des accidents vasculaires cérébraux (AVC). En l'absence de données locales, nous avons évalué la prise en charge de l'hypertension artérielle chez les patients suivis en neurologie pour un AVC.

**Méthodologie:**

Nous avons réalisé une étude rétrospective descriptive et analytique portant sur les patients hypertendus hospitalisés pour un AVC. Ces patients ont été évalués à M1, M3, M6 et M12.

**Résultats:**

141 patients ont été inclus, avec un âge moyen de 61 ans. La quasi-totalité (94,3 %) des patients bénéficiaient d'une bithérapie antihypertensive associant majoritairement un inhibiteur de l'enzyme de conversion et un diurétique (70,2 %). Au cours du suivi, 76 patients ont été évalués à M1, 50 à M3, 44 à M6 et 42 à M12. Le coût moyen du traitement antihypertenseur était de 13 771 francs CFA (21 euros) par mois. Les veufs, les patients sans profession, ceux à faible niveau d’étude et sans assurance maladie étaient inobservants au traitement antihypertenseur. À un an de suivi, la pression artérielle était contrôlée chez 80 % des 42 patients encore présents. Le non-contrôle de la pression artérielle était en rapport avec une mauvaise observance thérapeutique (p < 0,05).

**Conclusion:**

Cette étude a révélé des problèmes de suivi des patients hypertendus en post-AVC avec un nombre élevé de perdus de vue. La pression artérielle était contrôlée chez les patients régulièrement suivis et observants au traitement antihypertenseur.

## Abstract (en)

**Introduction:**

Reducing blood pressure after stroke is important to prevent recurrent stroke, but we have no data about the control of blood pressure in our context. The purpose of this study was to assess management of hypertension among post-stroke patients in a neurology department.

**Method:**

It was a retrospective study involving hypertensive stroke patients. They were followed up at 1, 3, 6 and 12 months after discharge.

**Results:**

141 patients fulfilled the inclusion criteria. The mean age was 61 years. Almost all patients (94.3%) received a dual antihypertensive therapy combining mainly an ACE inhibitor and a diuretic (70.2%). During follow-up, only 76 patients were assessed at M1, 50 at M3, 44 at M6 and 42 at M12. The average monthly cost of antihypertensive treatment was 13,771 CFA francs (21 euros). Non-adherence to antihypertensive medication were mostly noted in widows, patients without occupation, those with low education and no health insurance. At one year, blood pressure was controlled in 80% of the 42 patients still present. Non-control of blood pressure was related to poor therapeutic compliance (p<0.05).

**Conclusion:**

This study highlights follow-up issues in hypertensive post-stroke patients with a high number of lost to follow-up. Blood pressure was controlled in patients who were regularly followed and adherent to antihypertensive treatment.

## Introduction

L'hypertension artérielle (HTA) constitue le principal facteur de risque des accidents vasculaires cérébraux (AVC) [8, 19]. Une augmentation de 20 mmHg de la PAS augmente le risque de survenue d'un infarctus cérébral de l'ordre de 35 % et de 44 % le risque d'hémorragie cérébrale [26]. L'impact de l'HTA sur les AVC est important en Afrique subsaharienne. En effet, l'hypertension artérielle y constitue le principal déterminant des AVC ischémiques et hémorragiques [17, 28] et elle est responsable de l'augmentation significative des AVC dans cette région du monde [21]. En outre, on estime qu'en Afrique subsaharienne, le contrôle de la pression artérielle constitue un élément clé de la lutte contre les AVC [23]; d'autant plus que 46 % des Africains de plus de 25 ans sont hypertendus et exposés à un risque élevé d'AVC [7]. En prévention secondaire des AVC, l’étude PROGRESS a clairement démontré l'effet bénéfique des antihypertenseurs aussi bien chez le sujet hypertendu ou en cas de pression artérielle normale [25]. En dépit des efforts massifs et coûteux pour dépister et traiter les hypertendus, moins d'un tiers étaient effectivement contrôlés en France en 2015. Ce taux chutait même en dessous de 20 % chez les femmes [24]. En Afrique, les études publiées font le même constat. Une méta-analyse publiée en 2014 qui a inclus plus de 110 000 patients avec un âge moyen de 40 ans a déterminé que seulement 18 % des sujets hypertendus étaient traités en Afrique subsaharienne et 7 % d'entre eux avaient une HTA contrôlée [2]. Une récente publication de 2021 a démontré que dans les pays à revenus faibles et moyens, seulement un tiers des hypertendus avaient connaissance de leur maladie et seulement 8 % d'entre eux étaient contrôlés [29]. La conséquence est un excès du risque cardio-vasculaire en général et des AVC en particulier chez les populations noires africaines en lien avec cette HTA non contrôlée [30].

Les causes du non-contrôle de la pression artérielle sont multiples. Certaines ont trait au système de soins, d'autres aux patients eux-mêmes, notamment le défaut d'observance des traitements. Les dernières raisons impliquent parfois directement les médecins qui n'instaurent pas ou n'augmentent pas le traitement de façon appropriée alors que les objectifs thérapeutiques recommandés ne sont pas atteints; on parle d'inertie thérapeutique [5]. En Côte d'Ivoire, les seules données disponibles concernent l'observance des traitements antihypertenseurs en prévention primaire, avec des taux de contrôle globalement satisfaisants de 54 et 77 % dans deux travaux différents réalisés à Abidjan [14, 18]. Nous avons réalisé cette étude afin de faire l’état des lieux de la prise en charge de l'HTA en prévention secondaire des AVC dans un service de neurologie générale.

## Méthodologie

Nous avons mis en place une étude rétrospective, descriptive et analytique, portant sur les patients hospitalisés pour un AVC au service de neurologie du CHU de Bouaké, au centre de la Côte d'Ivoire, du 1^er^ janvier au 31 décembre 2020. Les patients décédés en cours d'hospitalisation n'ont pas été inclus. Les différentes variables étudiées concernaient les données sociodémographiques (âge, sexe, lieu de résidence, statut matrimonial, profession, assurance maladie, niveau d'instruction), les facteurs de risque vasculaire, le type d'AVC, le traitement antihypertenseur à la sortie du patient (nature du traitement, classe thérapeutique, nombre de prises quotidiennes, coût du traitement, posologie, coût total des médicaments) et le suivi des patients à 1, 3, 6 et 12 mois. Au cours des différents rendez-vous de suivi, on précisait le nombre de patients présents, les chiffres tensionnels obtenus (pression artérielle systolique (PAS) et pression artérielle diastolique (PAD)), l'observance thérapeutique, les effets secondaires éventuels, un changement du traitement antihypertenseur, une augmentation de la posologie, un arrêt du traitement par le médecin, une récidive d'AVC ou un décès. L'observance thérapeutique était appréciée par l'interrogatoire direct du patient ou d'un aidant. Elle était définie par le nombre de doses effectivement prises divisé par le nombre de doses prescrites sur la période de suivi et multiplié par 100. Ce taux encore, appelé proportion de jours couverts par le traitement, permet de définir une bonne observance pour des taux au-delà de 80 %. L'observance thérapeutique était considérée comme bonne au-dessus de 80 % et mauvaise en dessous de 80 %. Les patients perdus de vue étaient d'emblée classés dans la catégorie des non-observants. Le contrôle tensionnel était affirmé pour des chiffres de pression artérielle en dessous de 140/90 mmHg. Les données descriptives ont été exprimées en proportion ou en moyenne ± écart type. Le test de Chi2 et le test de Fischer (p < 0,05) ont été utilisés pour l'analyse des données.

## Résultats

Durant la période d’étude, 141 patients ont été admis pour un AVC dont 125 cas confirmés à l'imagerie. Parmi ces derniers, on enregistrait 75 % d'infarctus cérébraux contre 25 % d'hémorragies. L’âge moyen était de 61 ans avec des extrêmes de 24 et 90 ans. Le sexe ratio était de 1,2. La majorité des patients (95 %) ne disposaient pas d'une assurance maladie et plus de la moitié (54 %) étaient illettrés. La sédentarité (62 %) et l'HTA (59,6 %) représentaient les principaux facteurs de risque vasculaire. Les caractéristiques sociodémographiques et cliniques sont regroupées dans le Tableau I. À la sortie de l'hôpital, la quasi-totalité (94,33 %) des patients bénéficiaient d'une bithérapie antihypertensive associant majoritairement un inhibiteur de l'enzyme de conversion (IEC) et un diurétique (70,2 %), un IEC et inhibiteur calcique (20,6 %). Le coût moyen du traitement antihypertenseur était de 13 771 francs CFA (21 euros) par mois avec des extrêmes de 2 165 et 21 120 FCFA (3 et 32 euros). Quand on y rajoute le traitement des autres comorbidités, le coût médian de l'ordonnance passait à 22 573 francs CFA (34 euros) mensuellement pour des extrêmes de 11 805 à 34 125 FCFA (18 à 52 euros). Le Tableau II résume les paramètres de suivi. Les facteurs influençant l'observance thérapeutique et le contrôle des chiffres tensionnels sont relevés respectivement dans les Tableaux III et IV.

**Tableau I:** Caractéristiques sociodémographiques et cliniques Sociodemographic and clinical characteristics

Variables	n (%) n = 141
Âge (ans)	
≤ 40	13 (8,6)
]40-60]	50 (34,8)
]60-80]	72 (51,1)
> 80	6 (5)
Sexe	
homme	77 (54,6)
femme	64 (45,4)
Profession	
secteur informel	58 (41,1)
salarié	10 (7,1)
retraité	12 (8,5)
sans profession	61 (43,3)
Statut matrimonial	
célibataire	3 (2,1)
en couple	103 (73,1)
divorcé	2 (1,4)
veuf	33 (23,4)
Niveau d'instruction	
analphabète	77 (54,6)
primaire	33 (23,4)
secondaire	26 (18,4)
universitaire	5 (3,6)
Résidence	
Bouaké	70 (49,7)
hors Bouaké	71 (50,4)
Assurance maladie	
oui	7 (5)
non	134 (95)
Prise en charge	
patient lui-même	81 (57,5)
tierce personne	60 (42,6)
Nombre d'antihypertenseurs	
un	7 (5)
deux	133 (94,3)
trois	1 (0,7)
Nombre de prises quotidiennes	
une fois	2 (1,4)
deux fois	139 (98,6)
Coût mensuel des anti-HTA (FCFA)	
< 10 000	5 (3,6)
[10 000-15 000]	119 (84,4)
> 15 000	17 (12,1)

**Tableau II:** Paramètres de suivi des patients Patients monitoring parameters

Paramètre		MO	M1	M3	M6	M12
Patients présents		n = 141	n = 76	n = 50	n = 44	n = 42
Évolution PAS (mmHg)	PAS ≥ 140 n (%)	116 (82,2)	47(61,9)	25(50,0)	13(29,5)	8(19,0)
Évolution PAD (mmHg)	PAD ≥ 90 n (%)	73(51,7)	15(19,7)	11(22,0)	7(16,0)	4(9,5)
Niveau de bonne observance globale (n = 141) n (%)		-	54 (38,3)	41(29,0)	38(26,9)	37(26,2)
Niveau de bonne observance chez les patients présents aux consultations n (%)		-	54(71,0)	41(82,0)	38(86,3)	37(88,1)
Taux de contrôle de PA (%)		-	28(36,8)	22(44,0)	3068,1	33(78,6)
Taux de récidive d'AVC n (%)		-	2 (2,6)	1(2,0)	1(2,3)	1(2,4)
Taux de décès n (%)		-	3(3,9)	1(2,0)	1(2,3)	1(2,4)

**Tableau III:** Facteurs influençant l'observance thérapeutique selon la période d’étude Factors influencing therapeutic compliance according to the study period

Facteur	M1	M3	M6	M12
Âge	p = 0,958	p = 0,752	p = 0,252	p = 0,236
Sexe	p = 0,230	p = 0,457	p = 0,705	p = 1
Secteur d'activité	p = 0,009	p = 0,001	p = 0,006	p = 0,004
Domicile	p = 0,388	p = 0,711	p = 0,850	p = 0,570
Niveau d’étude	p = 0,005	p = 0,014	p = 0,001	p = 0,003
Statut matrimonial	p = 0,020	p = 0,002	p = 0	p = 0
Assurance maladie	p = 0,013	p = 0	p = 0	p = 0,002
Antécédent d'HTA	p = 1	p = 0,807	p = 0,615	p = 0,615
Antécédent d'AVC	p = 1	p = 0,319	p = 0,294	p = 0,294
Nombre de prises quotidiennes d'antihypertenseurs	p = 1	p = 1	p = 1	p = 1
Coût des antihypertenseurs	p = 1	p = 0,558	p = 0,847	p = 0,847
Coût total des médicaments	p = 0,314	p = 0,294	p = 0,368	p = 0,364
Type d'antihypertenseur	p = 0,648	p = 1	p = 1	p = 1
Récidive d'AVC	p = 0,286	p = 1	p = 1	p = 1
Effets secondaires	p = 0,371	p = 0,488	p = 1	p = 1

**Tableau VI:** Facteurs influençant l'atteinte de l'objectif tensionnel selon la période de suivi Factors influencing the achievement of blood pressure goal according to the follow-up period

Facteur	M1	M3	M6	M12
Âge	p = 0,312	p = 0,770	p = 0,926	p = 1
Sexe	p = 0,017	p = 0,559	p = 0,738	p = 0,714
Secteur d'activité	p = 0,181	p = 0,115	p = 0,524	p = 0,249
Domicile	p = 1	p = 0,153	p = 0,521	p = 0,269
Niveau d’étude	p = 0,190	p = 0,648	p = 0,031	p = 0,141
Statut matrimonial	p = 0,106	p = 0,285	p = 0,185	p = 1
Assurance maladie	p = 0,250	p = 1	p = 0,401	p = 1
Antécédent d'HTA	p = 0,132	p = 0,773	p = 0,738	p = 0,692
Antécédent d'AVC	p = 1	p = 0,121	p = 0,540	p = 1
Nombre de prises quotidiennes d'antihypertenseurs	p = 1	p = 1	p = 1	p = 1
Coût des antihypertenseurs	p = 0,774	p = 0,126	p = 0,448	p = 1
Coût total des médicaments	p = 0,007	p = 0,015	p = 0,068	p = 0,100
Type d'antihypertenseur	p = 0,436	p = 0,267	p = 0,176	p = 0,05
Récidive d'AVC	p = 1	p = 1	p = 0,002	p = 1
Effets secondaires	p = 1	p = 1	p = 1	p = 1
Observance thérapeutique	P < 0,0001	p = 0,001	p = 0,004	p = 0,001

## Discussion

Cette étude révèle un taux de contrôle de la pression artérielle globalement satisfaisant chez les victimes d'AVC régulièrement suivis en neurologie et qui étaient observants au traitement antihypertenseur. Les bithérapies antihypertensives ont constitué les traitements les plus prescrits chez ces patients à haut risque vasculaire. Cette première étude ivoirienne évaluant la pression artérielle en prévention secondaire des AVC comporte certaines limites, notamment un taux élevé de perdus de vue (141 patients à l'inclusion contre 42 à 12 mois de suivi). Aussi, le test utilisé pour mesurer l'observance au traitement était une adaptation de celui proposé dans la littérature qui classait l'observance en trois niveaux alors que dans notre étude, nous l'avons classée en seulement deux niveaux à savoir une bonne et une mauvaise observance. Enfin, la méthode d'observance utilisée, reposant sur des questions à réponses directes, comporte un risque de surestimation de l'observance en raison notamment des biais de mémoire. En procédant à une analyse plus détaillée des résultats, on constate une diminution progressive du taux de suivi des patients malgré les dispositions prises pour préciser la date exacte de leur prochaine consultation. En effet, sur 141 patients initialement recensés, 53 % (76 patients) ont consulté un mois après leur sortie de l'hôpital. Ce taux est passé à 35 % (50 patients) à 3 mois avant de stagner autour de 30 % soit 44 patients à 6 mois et 42 patients à 12 mois. L'absence aux consultations pourrait se justifier par plusieurs raisons. D'abord les difficultés financières éprouvées par les patients dont la majorité (43,2 %) n'avaient aucune activité professionnelle. Cette absence de source de revenu entraînait des difficultés à honorer les ordonnances quand on sait que le coût mensuel du traitement antihypertenseur était estimé à plus de 10 000 FCFA (15 euros). L’éloignement du site de consultation pourrait constituer un autre motif d'abandon. En effet, près de la moitié des patients initialement recensés (49,7 %) résidaient en dehors de la ville de Bouaké. Une autre raison reposerait sur les fausses croyances des patients qui ne verraient pas l'intérêt d'un suivi à long terme de la maladie hypertensive surtout après une certaine amélioration de leur état de santé. Cette dernière situation a également été décrite dans les pays développés, notamment en Chine où une étude publiée en 2018 a révélé que les fausses croyances constituaient une cause fréquente de mauvaise observance thérapeutique [6]. Parmi les patients présents aux différentes consultations de suivi, l'observance thérapeutique était globalement bonne et s'améliorait dans le temps, passant de 71 % à M1 à 88 % à M12. Par contre, rapportée à l'ensemble des patients initialement inclus dans l’étude (n = 141), l'observance restait moyenne variant entre 26 et 38 %. En dépit du bénéfice certain du traitement antihypertenseur sur la baisse de la pression artérielle, la mauvaise observance au traitement antihypertenseur après un AVC reste fréquente [4, 13]. Une étude au Zanzibar révèle un faible usage du traitement antihypertenseur chez les patients souffrant d'AVC, exposant ces derniers à un risque élevé de récidive [12].

Les facteurs de mauvaise observance identifiés dans notre étude étaient en rapport avec la profession, le niveau d’étude, le statut matrimonial et l'assurance maladie. En effet, la mauvaise observance prédominait chez les patients « sans emploi », dépourvus de revenu financier, qui éprouvaient des difficultés à honorer leurs ordonnances. La mauvaise observance est en général associée à des conditions socio-économiques difficiles [9, 22]. Ainsi, les patients qui ne disposaient pas d'une assurance maladie étaient inobservants au traitement. Adoubi *et al.* soulignent l'intérêt de l'assurance maladie dans l'observance thérapeutique des patients [1].

Le faible taux de couverture sociale dans notre étude (5 %) et dans la population ivoirienne en général a un impact sur la prise en charge des patients hypertendus dont le traitement moyen reste élevé, estimé au tiers du SMIG^1^1SMIG en Côte-d'Ivoire : 75 000 FCFA, soit environ 115 euros. La mauvaise observance était également mise en évidence chez les patients à faible niveau d'instruction, faute d'informations sur la gravité et les conséquences de l'hypertension [16, 22]. Dans la publication de Schutte *et al.,* la mauvaise observance du traitement anti-HTA était associée à la prise des traitements traditionnels en Afrique subsaharienne et au faible revenu des patients [29]. Des facteurs souvent rapportés tels que la survenue d'effets secondaires du médicament et le nombre élevé de prises journalières ne semblaient pas influencer l'observance thérapeutique de nos patients. Dans notre étude, les patients veufs étaient inobservants à leur traitement. Quand le conjoint est présent, son soutien moral aide ces patients, psychologiquement fragiles après leur AVC, à être plus observants à leurs différents traitements [4]. Concernant le niveau de contrôle de la pression artérielle, on notait une baisse progressive de la proportion des sujets hypertendus (PA ≥ 140/90 mmHg), passant de 81 % en début d’étude à 19 % à la fin. Soit, près de 20 % des patients qui n'avaient pas une pression artérielle contrôlée à un an. En dépit de l'intérêt du contrôle de la pression artérielle en prévention secondaire des AVC, plusieurs patients ont une pression artérielle non contrôlée en pratique clinique [10]. Une publication évaluant la pression artérielle chez les AVC en Afrique subsaharienne objective un niveau de contrôle faible autour de 40 % en lien avec un faible niveau d’éducation des patients et une mauvaise observance du traitement [31]. Une méta-analyse publiée en 2020 objective un taux de contrôle de la pression artérielle encore plus bas à 25 % chez les Africains victimes d'AVC [15]. L'observance thérapeutique constituait le seul facteur associé au contrôle de la pression artérielle dans notre étude. Cette association est bien décrite dans la littérature [31]. Pour d'autres auteurs, un mauvais contrôle de la pression artérielle était spécifiquement observé dans les hémorragies cérébrales [3]. Cette association n'a pas été analysée dans notre travail. Une récente publication de la République démocratique du Congo confirmait le rôle d'une pression artérielle élevée, donc non contrôlée dans la survenue des hémorragies cérébrales [17]. Sarfo *et al.,* dans une étude menée au Ghana et en Floride aux États-Unis, relevaient un taux élevé d'HTA résistante à 42 % en post-AVC chez les indigènes africains [27]. Ce taux élevé d'HTA résistante pourrait expliquer la difficulté à contrôler la pression artérielle des Africains noirs en post-AVC.

La plupart de nos patients (70,2 %) étaient sous une association fixe comportant un inhibiteur de l'enzyme de conversion et un diurétique thiazidique comme dans l’étude PROGRESS [25]. Cette étude randomisée en double aveugle a objectivé une réduction de 28 % du risque de récidive d'AVC chez les patients traités par IEC (Périndopril) associé à un diurétique (Indapamide) par rapport à ceux mis sous placebo. Les recommandations africaines et internationales proposent l'association d'un inhibiteur calcique à un bloqueur du système rénine-angiotensine en première intention pour traiter l'hypertension artérielle du sujet noir africain, ou même l'association d'un inhibiteur calcique à un apparenté thiazidique [11, 20]. Mais ces recommandations n’étaient pas spécifiques aux patients atteints d'AVC.

## Conclusion

Cette étude préliminaire a objectivé des résultats intéressants dans l’évaluation de la prise en charge de l'hypertension en post-AVC. Elle a en effet identifié des problèmes de suivi chez la majorité des patients et a mis en évidence un taux de contrôle correct de la pression artérielle chez les patients régulièrement suivis et observants à leur traitement antihypertenseur. La mise en place de programmes de suivi impliquant d'une part les équipes médicales pour la sensibilisation des patients et des aidants pour leur soutien psychologique envers les patients, et d'autre part l’État et les entreprises privées pour la vulgarisation et l'efficacité de l'assurance maladie, contribuerait à réduire le taux de perdus de vue et améliorer l'observance des patients hypertendus.

## Contribution des auteurs

Kouamé-Assouan AE, Gnazégbo A : conception de l’étude.

Kouamé-Assouan AE, Gnazégbo A, Sylla A : rédaction et correction du protocole et du manuscrit.

Bony KE, Karidioula HA : réalisation de l'analyse statistique.

Koffi YT, Touré A, Koné BK : collecte des données.

Kouamé-Assouan AE, Bony KE, Gnazégbo A : supervision, validation du manuscrit.

## Liens d'intérêt

Les auteurs ne déclarent aucun lien d'intérêt.
